# Supported Decision Making in South America: Analysis of Three Countries’ Experiences

**DOI:** 10.3390/ijerph18105204

**Published:** 2021-05-13

**Authors:** Alberto Vásquez Encalada, Kimber Bialik, Kaitlin Stober

**Affiliations:** 1 Sociedad y Discapacidad (SODIS), Miraflores, Lima 15046, Peru; 2 Inclusion International, London SE11 5RR, UK; 3 Department of Disability and Human Development, University of Illinois at Chicago, Chicago, IL 60608, USA

**Keywords:** supported decision making, legal capacity, guardianship, intellectual disability, psychosocial disability, South America

## Abstract

*Background*. Following the adoption of the UN Convention on the Rights of Persons with Disabilities, there has been increased interest in supported decision making (SDM) as a strategy to realize the right to legal capacity of persons with intellectual and psychosocial disabilities. Support for decision making has been delivered formally through SDM services as well as informally through interpersonal networks. Various SDM programs have made efforts to systematize informal support, showcasing a variety of SDM delivery models that could benefit SDM implementation in low- and middle-income countries. *Methods.* This article examines and discusses three SDM projects in South America (Colombia, Peru, and Argentina) that have been directly implemented by civil society organizations, including organizations of persons with disabilities and their families. Analyzed program components include person-centered planning, the nature of support relationships, the presence of supporter training, community involvement, and the utilization of quality assurance measures such as monitoring and program evaluation. *Conclusions.* The results and learning from these initiatives constitute a valuable source of information for legislators and policymakers for the future development of supported decision-making programs, which are an essential form of support and a mechanism for fulfilling the right to legal capacity in low resource settings.

## 1. Introduction

Interest in supported decision making has increased significantly following the adoption of the United Nations Convention on the Rights of Persons with Disabilities (CRPD). Article 12 of the CRPD acknowledges the universal right of persons with disabilities to enjoy legal capacity on an equal basis with others in all aspects of life [1]. It also sets out an obligation to provide persons with disabilities with access to support in the exercise of their legal capacity, which is subject to proportional and tailored safeguards. This recognition has prompted renewed interest in introducing policy and legislation to rectify the historical denial of legal capacity from people with intellectual and psychosocial disabilities. In this context, supported decision making (SDM) has been conceptualized as an alternative to guardianship [2], which allows individuals with disabilities to make decisions about their own lives with support.

### Supported Decision Making as a Means to Exercise Legal Capacity

Throughout history, persons with disabilities have been denied or have had restrictions placed on their right to legal capacity in different areas. These limitations have been commonly justified on the basis of the diagnosis of an impairment (status approach), previously made decisions and their outcomes (outcomes approach), or deficient decision-making skills (functional approach) [3]. The Committee on the Rights of Persons with Disabilities has stressed that each of these three approaches to the removal of legal capacity are a violation of the human rights of persons with disabilities [4]

From a rights-based perspective, guardianship and other forms of substitute decision making represent a violation of the right to equal recognition before the law and, more specifically, the right to legal capacity, which is necessary for the enjoyment of all other rights [5]. Even substitute decision-making regimes that purport to protect an individual’s best interests still restrict individuals from exercising self-determination and enjoying the dignity of risk. Beyond these criticisms, guardianship does not promise protection for an individual with disabilities, and there are accounts of substitute decision makers taking advantage of, ignoring the explicit wishes of, or abusing the individual with a disability they were appointed to represent [6,7]. In the United States, the National Council on Disability recently highlighted exploitation and abuse by guardians as well as the failures of the court systems to address these issues [8].

The adoption of the CRPD in 2006 represented a paradigm shift away from substitute decision making, largely due to the inclusion of Article 12.2, which affirms that “persons with disabilities enjoy legal capacity on an equal basis with others in all aspects of life” [1]. Furthermore, Article 12.3 of the UNCRPD explicitly calls for the provision of SDM, asserting that “states parties shall take appropriate measures to provide access by persons with disabilities to the support they may require in exercising their legal capacity” [1]. With this article, the UNCRPD transformed SDM as an alternative to guardianship from an ideal to a human rights imperative [9]. Despite outlining their necessity, the UNCRPD does not provide specific details on how such support should be provided [10].

While there is no universal conceptualization of SDM [2], the term is commonly used to refer to both the practical process and legal recognition of the practice [11,12,13]. The concept of SDM originated in Canada, prior to the CRPD, as a means to break down barriers which prevented members of the disability community from becoming self-determined citizens [9,11]. The Canadian Association for Community Living, now named Inclusion Canada, authored the first “principles” of SDM in their report on alternatives to guardianship in 1992; it was these principles that distinguished SDM, the process specifically linked to legal capacity, from mere support for making decisions [11].

Despite this early attempt to distinguish SDM as a purposeful, principle-based practice, SDM remains an elusive concept, having been adopted and appropriated in various ways [2,9,10,11,14]. Legally recognized supported decision-making regimes vary significantly from country to country [15,16]. Different models of SDM include formal and informal networks, support agreements, independent advocates, peer support, advance directives, and personal assistance, among others, which provide a very broad range of support to individuals—from access to information and support for communication to empowerment and relationship building [5].

SDM can be delivered through a variety of formats, both as formal service provision through agencies and organizations or on an informal basis through interpersonal networks. On a project to project level, the differences in SDM delivery between varying projects can be attributed to differences in the needs of the participating persons with disabilities [14]. Similarly, the area of life for which a person is accessing decision-making support impacts the way this support is administered. For instance, personal life decisions, health decisions, and financial decisions all require varying levels of formality and the documentation of decision-making support. Models for the delivery of SDM also vary based on the social, economic, and cultural context of the communities in which it is being practiced. In low- and middle-income countries with fewer resources and less availability of formal service providers, SDM and other supports tend to be delivered directly by families and through support from communities. However, the extent to which decisions of people with disabilities are respected is typically proportionate to the degree that disability is or is not stigmatized in that community [17].

SDM tools and resources that support families and service providers to implement SDM also vary based on local standards for accessible information (including plain language, easy-to-read, and other formats) and the availability of assistive technologies individuals may use as communication supports [17], both of which tend to be less available in low-resource settings. In some cases, cultural beliefs that emphasize respect based on age and gender may also impact access to SDM, with decreased access to SDM for younger people, women, and young women in particular documented in low-resource contexts, including Kenya, India, and Lebanon [17].

While there is no agreement on a singular conceptualization of SDM that is applicable across all global contexts, there is empirical consensus that additional research on the implementation and impact of SDM is needed [18,19]. Bigby et al. noted that there is limited empirically oriented literature about the delivery or practice of SDM [20].

## 2. Methods

This article analyzes three supported decision-making projects in South America—Colombia, Peru, and Argentina—which have been directly implemented by civil society organizations, including organizations of persons with disabilities and their families (OPDs). The Colombia-based SDM project was first identified in a wider exploration of SDM pilots and initiatives around the world that were reviewed by the authors in preparation for the UN Special Rapporteur on the rights of persons with disabilities’ thematic report on legal capacity [5]. This initial search utilized academic databases, JSTOR and ProQuest, as well as general search engines to find the “grey literature” such as programmatic reports that were not published in academic journals. The SDM projects in Argentina and Peru were identified at a later date by the first author through Spanish-language searches on SDM in South American countries.

These three programs were selected for review and consideration in the present paper because of the limited empirical attention paid to SDM in South American countries, despite their advances in legal capacity reform. In addition, these SDM projects provided an opportunity to analyze SDM in low- and middle-income countries in light of the overrepresentation of SDM models from high-income countries in the literature. However, this paper does not aim to present a comprehensive overview of all existing SDM pilots or SDM programs in the region. It is likely that many organizations or associations offering SDM programs or services do not have published information about such programs if they do not have websites, are not required to publish reports per funding agreements, or are in early stages with limited data.

While some jurisdictions within the region have implemented legislation to govern SDM, including Argentina, Brazil, Colombia and Peru, the exploration of legislative strategies falls outside the scope of this paper.

### 2.1. SDM Cases from South America

#### 2.1.1. Colombia

In Colombia, the pilot project “Supported Decision Making and Community Life in Colombia, under Article 12 and 19 of the Convention on the Rights of Persons with Disabilities” was implemented between February 2015 and December 2016, as part of a collaboration between Asdown Colombia, Nodo Comunitario de Salud Mental and PAIIS (Programa de Acción por la Igualdad y la Inclusión Social), and with funding from the Open Society Foundations. This project was conducted with a participatory action research framework and was grounded in person-centered planning. It included 23 adult participants, 12 with intellectual disabilities and 11 with psychosocial disabilities, from six localities within the city of Bogota. The selection of the participants was based on home-based interviews and quick participatory assessments.

The pilot aimed at building a personalized support system designed to contribute to capacity building for decision making and the encouragement of independent living. This support system also sought to improve relationships with close relatives, the extended family and the community. For that purpose, the research team established relationships with participants and their family members and identified communication and support needs. Then, the participant and their family developed a map of networks that the person could use for support. Participants also engaged in a “Projection of a Life Plan” project that contributed to the creation of their personalized booklet. This allowed them to describe their goals, dreams, and basic preferences. Information on support for community life, autonomy, and daily living were also included in the booklet. Family members also participated in training to learn about providing support. Participants and their families were able to evaluate goals based on the life plan created [21].

This project evaluation reported that most participants lacked the autonomy for decision making prior to this project [21]. The participants with intellectual disabilities were not empowered to engage in decision making in daily life, and the participants with psychosocial disabilities were found to be isolated, with low participation in the community. Families were also found to be the main support for people with disabilities, whether intellectual or psychosocial, with limited capacity to foster autonomy and independence. In addition, most families reported that legal services had recommended they resort to guardianship. Qualitative interviews and observation revealed that some participants were empowered through the pilot program to make their voices heard, and that families adjusted their approach to support. However, there was minimal information provided about the systematization of this program and the findings provided were not substantial. Decisions with legal implications were not addressed in the report.

It is important to note that this pilot was developed prior to the legal reform reached by Colombia in 2019, which eliminated guardianship from the Colombian legal system and replaced it with regimes for supported decision making [22].

#### 2.1.2. Peru

From 2016 to 2018, Sociedad y Discapacidad (SODIS) and the Peruvian Down Syndrome Society (SPSD), implemented the pilot project “Support Networks for Decision Making and Community Life” as part of a set of strategic actions aimed at achieving legal capacity reform, which was finally achieved in September 2018 when the Peruvian government passed the Legislative Decree No. 1384. The project was funded by the Inter-American Foundation, with additional support from the Open Society Foundations. Its main goal was to promote decision-making support networks for people with intellectual and psychosocial disabilities in order ensure they could exercise of their rights to legal capacity and to live independently in the community. The pilot included 20 adult participants from Lima, 10 with intellectual disabilities and 10 with psychosocial disabilities, as well as their respective family members and other community actors identified as potential supporters.

The project involved interventions at three levels. At an individual level, the facilitating team implemented person-centered planning activities and individual counselling. At an interpersonal level, they developed group workshops, peer support meetings, and family meetings. At a community level, stakeholder mapping and outreach activities with community-based organizations were conducted. All interventions were developed separately for people with intellectual and psychosocial disabilities. They were carried out in partnership with public entities, the Social Security Health System (EsSalud) and the community mental health center of Alto San Gabriel.

This project benefitted from an external evaluation. This evaluation included semi-structured interviews with participants and families and networks, as well as the facilitating team [23,24]. While participants with intellectual disabilities generally reported high satisfaction with the activities and increased autonomy, they faced challenges in achieving their person-centered goals, particularly due to lack of effective support. Participants with psychosocial disabilities, who attended activities less regularly, found significant value in peer support strategies, but framed them as “therapeutic” [25]. In both groups, decisions with legal implications were limited and the adequate involvement of community actors was not achieved. However, empowerment through the improved knowledge of rights was noted.

The evaluation highlights the importance of exploring different flexible strategies based on identified support needs, barriers and networks, as well as incorporating people with disabilities themselves as co-facilitators [23,24].

#### 2.1.3. Argentina

In Argentina, the National University of Mar del Plata carried out the pilot project “Persons with Disabilities, the exercise of their legal capacity and decision making: implementation of supports in different contexts” from February 2017 to April 2019. This project aimed to identify and critically analyze support systems for decision making based on the daily experience of participants and was conducted with a participatory action research framework. The project was implemented in partnership with local institutions, such as the segregated school “C.I Alito” for persons with intellectual disabilities, the Down Syndrome Association of Mar del Plata, the Municipal Mental Health Centre, and the Mental Health Centre “Proyecto Be”. They also had financial support from the Open Society Foundations. The project involved 16 adult participants, 8 with intellectual disabilities and 8 with psychosocial disabilities. Some participants had “therapeutic companions,” which are paid health workers who support vulnerable individuals for improvement in their recovery and quality of life, often judicially appointed [26].

Similar to the previous projects discussed, this project used a person-centered planning methodology, which included tools such as genograms, ecomaps, and network maps [27]. From those exploratory individual semi-structured interviews and group workshops, participants identified support individuals and networks in their daily life, as well as their limitations. Participants with psychosocial disabilities responded positively to individual approaches, and some benefited from spaces for group reflection to identify, sustain, and build on the supports of everyday life. Participants with intellectual disabilities required a more active involvement of families. Decisions involving treatment, sexuality, independent living, and patrimony were discussed by many participants.

The evaluation identified the need to support a flexible and dynamic “toolbox”, co-built with people with disabilities themselves. Likewise, the role of organizational structures in the design of supported decision making was stressed [27].

## 3. Discussion

This section discusses program components that were identified as recurring among the examined SDM pilot projects. Program design elements, strategies used, and limitations across projects are discussed. Due to program evaluations being small-scale, the available information proves insufficient to draw conclusions about the effectiveness of individual elements of the programs. As a result, the following discussion does not present a single ideal program structure, but rather offers insight into a range of structural elements and approaches and potential strengths and limitations.

Notably, the three experiences described were grounded in the CRPD. The CRPD, and in particular the CRPD Committee’s interpretation of article 12 [4], served to define the conceptual frameworks and intervention principles of the projects. There are specific references to the CRPD and the Committee’s work in all project documents [21,26,28,29]. Voluntariness, respect for will and preferences, and sensitivity to diverse support needs were taken as guiding principles for supported decision making. Additionally, attention was given to general accessibility measures and reasonable accommodation following the CRPD Committee’s General Comment. This confirms the momentum created by the CRPD for the development of SDM practice [9].

### 3.1. Strategies

Person-centered planning (PCP) was a recurring structural element in all three projects. PCP is a life planning strategy centered on the will and preferences of an individual with intellectual disabilities, and allows service providers to support decisions and actions that will help the individual to achieve their goals [30]. PCP has been found to be effective in improving the life experiences of people with intellectual disabilities [31,32], and the broader philosophy and principles of PCP are also informing person-centered approaches in mental health provision [33]. Various SDM projects around the world use person-centered approaches [34,35,36] and the European Association of Service Providers for Persons with Disabilities (EASPD) recommended it as a central tenet for SDM [37]. For example, PCP has been conceptualized as a potential safeguard against instances of coercion, as paternalistic attitudes and pressure on supporters to take the preferences of family members and friends into account when providing support have been reported [38].

All three analyzed projects used elements of PCP as a strategy. In Colombia, the project used a wide variety of PCP tools that were adjusted to the needs of the participants and their families. Each participant was given five (5) sessions with a duration of approximately 2.5 h to engage with the life planning tools. In Peru, participants were expected to develop and implement a person-centered plan with specific objectives and activities. However, this aim was later abandoned with the group of people with psychosocial disabilities in favor of less structured approaches for identifying goals and supporters. Of the 10 participants with intellectual disabilities, eight designed a person-centered plan during the project. While there is little information about how PCP was applied in the Argentinean experience, the project reported using PCP tools including genograms, ecomaps, and network maps.

While PCP is commonly utilized in SDM work, the conflation of SDM and PCP can be potentially problematic. While PCP can be a useful tool for supporters to engage with during the implementation of SDM, PCP as a philosophy must be clearly distinguished from more concrete strategies for SDM. There is a risk of turning support into a tick-box exercise; that is, reducing support—a complex process—into procedures and rules which are task-focused. Moreover, to be successful, PCP requires skillful facilitation, the availability of support and services, intensive training, and a shift in perceptions [39], which may be challenging to implement in low-resource settings that lack access to PCP implementation resources and training.

### 3.2. Diversity of Participants

The three programs focused on both people with intellectual and psychosocial disabilities as their primary beneficiaries. Within such groups, the participant sample included men and women, people with and without guardianship, people with different education levels, and people with and without family support. However, none of the three programs included participants who had higher support needs. This limitation is not unique to South America—although supported decision-making strategies for people with profound intellectual and multiple disabilities (PMID) have been found to be successful [40,41] and tools have been created to guide supporters providing SDM to people with PMID [42], most SDM programs still tend to be limited to people who could be identified as having a mild intellectual disability [20]. SDM practice would benefit from additional examples of SDM programs including people with PMID, including in low- and middle-income countries.

While the pilot projects in Argentina and Colombia included persons with intellectual and psychosocial disabilities in the same activities, the Peruvian program worked separately with both groups, prioritizing group and peer support strategies for persons with psychosocial disabilities. This is again consistent with international SDM practice, where pilot projects vary in their inclusion of participants with different types of impairments [43,44].

Including participants with different types of disabilities and varying levels of support needs in pilot projects may better prepare organizations to provide support services to the diverse disability community and encourage greater individualization in the execution of SDM programming. However, because long-term results have not been reported from the discussed programs, it is difficult to ascertain how the results for individual participants are affected when the SDM programs focus on a specific constituent group versus the cross-disability community. The diversity of the disability community demands heterogenous support, and it is arguable that programs which focus on the needs of a specific group could be more effective than a singular model or “one size fits all” approach [5]; however, the models tested in these scenarios will be less widely scalable as a result. Further research is needed to understand the benefits of having identity group-specific SDM programs, versus programs inclusive of diverse disability groups.

### 3.3. Nature of Supporters

The relationship between an SDM user and supporter can be analyzed using two variables: the number of supporters, and the relationship between the decision maker and supporter. SDM programs often use either primarily single supporters or multiple supporters [45,46], although some may also use a hybrid structure that includes elements of both one-on-one support and group support [34,47]. SDM programs can also be divided into those that utilize an existing relationship of trust for the provision of support (“trusted supporter”) [36,48], and those that use a professional or trained supporter without a previously established relationship (“professional supporter”) [43,49] for the provision of support. Some projects also used hybrid models that involved both trusted and professional supporters [34,50].

Existing, trusted relationships have shown to be constructive because familiarity contributes a degree of comfort to the support relationship, and supporters already had a working knowledge of the SDM user and a level of trust that benefitted the decision-making process [51,52]. The use of trusted supporters is particularly relevant to the application of SDM in low-resource settings, which tend to lack the history of institutionalization exhibited in higher income countries. The decreased likelihood that individuals in low- and middle- income countries have experienced institutionalization means that people with disabilities in these contexts are less likely to have been separated from their family and community networks that can provide essential support for decision making as trusted supporters. In Latvia, a pilot program’s evaluation noted that using a trusted person as one’s supporter best replicated the natural decision-making processes in the larger community [34]. Despite these benefits, over reliance on supporters who have existing, trusting relationships with SDM users may also be problematic. Some people who could benefit from SDM may have no existing social network, which may be the case for individuals who have experienced life in institutional settings. Similarly, other potential SDM users may have experienced abuse and manipulation in relationships with family members and may therefore prefer a professional supporter [47]. Certain programs have responded to the barrier of social isolation by building trusting relationships with professional supporters [49].

The three programs explored in this paper used supporters from existing, trusted relationships within broader support networks identified from members of the community. In Peru, each participant with an intellectual disability selected a “support network.” They were almost always people known and close to the participants, such as family, friends, and neighbors. In some cases, because of the lack of commitment of the supporters, the responsibility for the most support activities was taken up by the main caregiver or other members of the participant’s family, some of whom felt burdened by complying with the PCP’s activities [23]. In the group of participants with psychosocial disabilities, supporters were mainly relatives, but they showed a lower level of commitment. In Colombia, there was also a focus on support networks, including support from community organizations, rather than individual supporters. However, there is not much information on how it was operationalized. The evaluation identified a lack of “circles and opportunities” to maintain friendships and loving relationships or to meet new people. Families were identified as “the engine and main support” for participants. It also notes that participants were “isolated from extended family and, therefore, their family support network ends up being diminished” [21]. In Argentina, supporters were identified within existing families and community networks, but there is no description on how this was conducted, or how supporters operated.

It should be noted that, due to the exploratory nature of the three experiences, the focus was placed on exploring support needs and identifying potential supporters, which did not allow sustained SDM work. However, it was noted that preconceived notions and paternalism may affect SDM relationships based on existing relationships. These limitations are not uncommon and may jeopardize the person-centered nature of SDM [34].

### 3.4. Training

Training for supporters can contextualize the role of the supporter and provide a rationale for the support they are providing. The training that supporters receive can influence a support relationship, and training that occurs in an ongoing, systematic fashion has been documented as a key factor in quality SDM practices [43].

Pilot programs in Colombia and Peru provided training to supporters and family members throughout the project. Family understanding and support can benefit SDM processes between a person with a disability and their family member supporter. The evaluation of the Peruvian project indicates, for example, that these training exercises were positively valued by family members as spaces for learning and reflection [53]. Some SDM pilot projects have recommended one-on-one training for supporters as a more effective model than facilitated group trainings [54], but this model may provide challenges to organizations in low- and middle-income countries that may have fewer financial and human resources to contribute to the delivery of SDM projects.

The Colombian pilot project also showed that training and access to resources on SDM does not need to be limited to the individual playing the support role, and opportunities can be provided for training individuals with disabilities in SDM strategies as well. This program developed training resources for participants, with the “Projection of a Life Plan” tool, which provided information on community life, autonomy, and daily living in a personalized booklet that participants could then use to detail their goals and preferences [21]. Ensuring that the individual receiving the decision-making support has also received training about their right to make decisions and the ways they can be supported to do so is an important strategy for ensuring the individual is empowered to be fully engaged in the process.

### 3.5. Community Involvement

When considering community attitudes towards SDM, it is important to note that historical discrimination against people with intellectual and psychosocial disabilities has contributed to a culture of exclusion and low expectations [55]. Hence, SDM programs should also consider community involvement to prevent instances of discrimination from occurring beyond the limits of the SDM relationship. As Devandas notes, providing support to make decisions is only helpful so long as those decisions are recognized by others [5]. Community involvement can help to address structural factors that can limit people’s autonomy, such as stigma, discrimination, and institutionalization.

Among all three projects analyzed, a specific objective of involving the community in the implementation of the project was a feature. In Peru, community organizations were identified and participated in reflection on the importance of SDM and on their own needs, resources, and possibilities for action [25]. The Colombian project reported a reinforcement of participation in local settings and community activities, which helped increase social contacts, friendships, and the expression of emotions [28]. These experiences also show the potential of partnering with other organizations and programs that provide services or support to people with disabilities.

### 3.6. Monitoring

The monitoring of SDM practice was not a strong feature of these three pilot programs, largely because involvement of the organization in the interactions between decision makers and supporters was limited. The Colombian project emphasized regular follow up, but the report did not offer clear, systematized methods [21]. For example, while parents received training on SDM strategies, there was no monitoring to see if these strategies were being utilized. Similarly, the Peruvian project reports a periodic monitoring of commitments of supporters but only as part of the PCP framework. In general, monitoring was based on the achievement of goals, but did not address the process by which choices were made [25,28].

These programs could benefit from monthly meetings with the decision maker to ensure the process is working. Monitoring not only collects data to inform the overall SDM movement but can help SDM programs identify challenges in their implementation and address them as they arise [43]. It is likely that preliminary and recurrent monitoring can support SDM to be practiced as intended, and ensure it is not recycling paternalistic tendencies of substitute decision making.

### 3.7. Evaluation

The three SDM programs in Peru, Colombia, and Argentina all made an effort to include evaluation as a component of the projects. The inclusion of an evaluation phase is significant because evaluation is uncommon among SDM programs, and the evaluation is often poor when it is included in SDM programs [20]. The projects in Argentina and Colombia were implemented with a participatory approach, although the scope of the approaches used is not specified. The evaluation of the pilot project in Peru, which used an independent evaluator, provided the most comprehensive overview. With the dearth of useful data on SDM programs, it would be beneficial to the field if both a participatory approach and external evaluation are considered in future SDM experiences.

The three programs analyzed used interviews and observation to collect data on project results. SDM, as a personalized and flexible tool, is well suited for qualitative studies. Qualitative data collection methods are beneficial because they allow evaluators to learn more about broader attitudes of participants in the program and their communities. They could also benefit the understanding of interactions between SDM programs and legal structures.

Evaluations did not include data on the cost of implementing SDM. Publishing details of a program’s operating budget appears to be a rarity among SDM projects, but this information is a necessary advocacy tool in encouraging governments and organizations to adopt their own pilot programs to begin the process of introducing these mechanisms in their jurisdictions [20]. Detailed cost data from the program level can be used in broader advocacy as well, potentially with regard to the move away from guardianship.

## 4. Conclusions

This article discussed three SDM experiences in South America across reoccurring variables and dimensions of SDM: strategies, participant diversity, the nature of support relationships, supporter training, the monitoring of SDM process, program evaluation, and the role of community advocacy. Building upon Bigby et al.’s work, this article highlights a greater need for data collection and evaluation, inclusivity in participant recruitment, effective safeguards, and additional pilot projects [20]. The flexibility of support arrangements, quality and consistent training for supporters, and advocacy measures are also shown as necessary. The structures and practices identified in this article also suggest that nuanced and individualized SDM programs can be beneficial in all global contexts, as ultimately, SDM models need to be structures that work for individuals.

When considering the application of SDM in the South American region, it is important to acknowledge that contextual factors likely played a role in SDM implementation. Building on the learning from other regions that pursue SDM, this exploration of delivery models in South America can help to refine conceptualizations of best practice in program design and its applicability across different contexts. Among the literature which primarily emphasizes learning from SDM projects in high-income countries, the South American experience indicates that SDM projects can be implemented successfully in low- and middle-income countries. Notably, the projects in Colombia, Peru, and Argentina made use of strategic partnerships between OPDs, other civil society organizations, and universities. This delivery-through-partnership model can be effective for cost-sharing and maximizing resources, which is particularly applicable to the implementation of SDM projects in low-income settings. Particularly in contexts where formal service delivery organizations do not exist, these strategic partnerships in low- and middle-income countries can fill gaps and ensure the availability of SDM resources where they otherwise may not exist. Moreover, the projects benefited from international cooperation, which highlights the critical role of international agencies and private donors in supporting innovative research and practices for the advancement of the rights of persons with disabilities, in line with Article 32 of the CRPD.

One significant limitation is the small scale of the practices analyzed, which is a major barrier to broader policy reform. Future SDM efforts should focus on the establishment of large-scale programs, long-term projects, the consideration of program costs, and the evaluation of what formal SDM services and programs do or should look like. Sequential projects could also be beneficial, as follow-up projects could correct shortcomings in initial projects.

Finally, it is not insignificant that these SDM projects have been carried out in countries that have achieved legal reforms in the legal capacity of people with disabilities. This may indicate that SDM efforts may contribute towards a transition away from substitute decision making and guardianship. While some proponents of SDM have argued that the practice can exist in tandem with substitute decision-making regimes [11], the continuation of those practices is contrary to the CRPD and the rights and inclusion of persons with disabilities. Given the legal capacity reforms achieved in some countries where guardianship has been abolished, future research efforts could contribute to exploring the functioning of SDM in these enabling legal frameworks.

## Data Availability

The data that support the findings of this study are available from the corresponding authors, upon reasonable request.
